# A “One-Health” approach for diagnosis and molecular characterization of SARS-CoV-2 in Italy

**DOI:** 10.1016/j.onehlt.2020.100135

**Published:** 2020-04-19

**Authors:** Alessio Lorusso, Paolo Calistri, Maria Teresa Mercante, Federica Monaco, Ottavio Portanti, Maurilia Marcacci, Cesare Cammà, Antonio Rinaldi, Iolanda Mangone, Adriano Di Pasquale, Marino Iommarini, Maria Mattucci, Paolo Fazii, Pierluigi Tarquini, Rinalda Mariani, Alessandro Grimaldi, Daniela Morelli, Giacomo Migliorati, Giovanni Savini, Silvio Borrello, Nicola D'Alterio

**Affiliations:** a Istituto Zooprofilattico Sperimentale dell'Abruzzo e Molise “G. Caporale”, Teramo, Italy; b Ospedale San Liberatore Presidio COVID-19 Atri, Teramo, Italy; c Direzione Sanitaria ASL, Teramo, Italy; d Reparto di Microbiologia e Virologia clinica, Ospedale Civile Spirito Santo, Pescara, Italy; e UOSD Malattie Infettive Ospedale G. Mazzini, Teramo, Italy; f UOC Malattie Infettive Ospedale SS Filippo e Nicola, Avezzano (L' Aquila), Italy; g UOC Malattie Infettive Ospedale S. Salvatore, L'Aquila, Italy; h Direzione Generale della Sanita' Animale e dei Farmaci Veterinari, Ministero della Salute, Roma, Italy

**Keywords:** SARS-CoV-2, COVID-19, Molecular characterization, Next generation sequencing, Mutations, Variants, One health, Veterinarians

## Abstract

The current pandemic is caused by a novel coronavirus (CoV) called SARS-CoV-2 (species *Severe acute respiratory syndrome-related coronavirus*, subgenus *Sarbecovirus*, genus *Betacoronavirus*, family *Coronaviridae*). In Italy, up to the 2nd of April 2020, overall 139,422 confirmed cases and 17,669 deaths have been notified, while 26,491 people have recovered. Besides the overloading of hospitals, another issue to face was the capacity to perform thousands of tests per day. In this perspective, to support the National Health Care System and to minimize the impact of this rapidly spreading virus, the Italian Ministry of Health involved the Istituti Zooprofilattici Sperimentali (IZSs), Veterinary Public Health Institutes, in the diagnosis of SARS-CoV-2 by testing human samples. The Istituto Zooprofilattico Sperimentale dell'Abruzzo e del Molise is currently testing more than 600 samples per day and performing whole genome sequencing from positive samples. Sequence analysis of these samples suggested that different viral variants may be circulating in Italy, and so in Abruzzo region. CoVs, and related diseases, are well known to veterinarians since decades. The experience that veterinarians operating within the Public Health system gained in the control and characterization of previous health issues of livestock and poultry including avian flu, bluetongue, foot and mouth disease, responsible for huge economic losses, is certainly of great help to minimize the impact of this global crisis.

## Highlights

• Italian VPH institutes (IZSs) are currently involved in SARS-CoV-2 diagnosis.
• IZSAM is processing 600 samples from Abruzzo region per day.
• 16 complete sequences were deposited on GISAID.
• Outbreaks in Abruzzo are related with those from northern Italy and northern Europe.
• Veterinarians play a crucial role in the diagnosis and characterization of SARS-CoV-2.

## 1 Introduction

The current pandemic caused by a novel coronavirus (CoV) called SARS-CoV-2 has been named by the World Health [1,2] Organization (WHO) as COVID-19. Even if 80% of COVID-19 human cases are mild, they can be still distressing and long-lasting. Most common symptoms of the infection are fever, dry cough, and shortness of breath. About 20% of infected patients may develop severe cases, and a small percentage of them (5%) may become critically ill. Patients with severe cases usually develop pneumonia or acute respiratory distress syndrome (ARDS), a condition that may require mechanical ventilation and intensive care unit treatment [3]. ARDS is often fatal [4]. The novel epidemic, recognized as a public health emergency of international concern on January 302,020, and acknowledged at a pandemic on March 112,020, was initially recognized in December 2019 in Wuhan City, Hubei Province, China, and continues to expand [5].

In Italy, up to the 8th of April 2020, overall 139,422 confirmed cases and 17,669 deaths have been confirmed, while 26,491 people have recovered (data source: National Department of Italian Civil Protection, available at: http://arcg.is/C1unv). Italian policy makers continue to urge people to stay at home and observe social distancing. Italy is experiencing more deaths than China, the country where the infection originated, which now officially reports 4,642 deaths. Since the infection was first identified in Codogno (Lombardy region) on February 21st, in less than three weeks, COVID-19 overloaded the National Health Care System (Servizio Sanitario Nazionale, SSN) in the northern Italy. It turned the hard hit Lombardy region into a grim glimpse of what countries may expect if they cannot slow down the spread of the virus and “*flatten the curve*” of new cases, which in turn allows treatment of sick patients without overloading the capacity of hospitals. Italy established draconian measures by restricting movement and closing all stores except for pharmacies, groceries and other social essential services. However, these measures did not come in time to prevent the surge of cases that has deeply taxed the capacity even of a well-regarded health care system.

SARS-CoV-2 belongs to the species *Severe acute respiratory syndrome-related CoVs* (*SARS-rCoV*) within the subgenus *Sarbecovirus*, genus *Betacoronavirus* together with SARS-CoV-1 strains from humans and SARS-rCoVs from wild carnivores and horseshoe bats (genus *Rhinolophus*) [2].

The virus harbors a linear single-stranded positive RNA genome of nearly 30 kb. At the very 5′-end of the genome is a leader sequence which is the unique characteristic in CoV replication and plays critical roles in the gene expression of CoV during its discontinuous sub-genomic replication [6]. Downstream, the 5′-most two-thirds of SARS-CoV-2 genome comprises the replicase gene, which consists of two overlapping open reading frames, ORF 1a and 1b translated to produce two large polyproteins, pp1a and pp1b. Cleavage of the replicase polyproteins is predicted to result in 16 end-products; nsp1–nsp11 encoded in ORF1a and nsp12–16 encoded in ORF1b [7]. Located downstream of ORF1b are four ORFs that code for structural proteins (spike (S), envelope (E), membrane (M) and nucleocapsid (N) proteins) and additional ORFs coding for accessory genes. As SARS-CoV-1, the S (through the S1) protein mediates viral attachment to the specific cell receptor angiotensin-converting enzyme type 2 (ACE2) [1] and fusion between the envelope and plasma membrane. As for other CoVs, the S protein is also the main inducer of virus-neutralising antibodies. The S protein of SARS-CoV-2 has a functional polybasic (furin) cleavage site at the S1–S2 boundary through the insertion of 12 nucleotides, which additionally lead to the predicted acquisition of three O-linked glycans around the site [8]. Six residues of the receptor binding domain (RBD) have been shown to be critical for binding to ACE2 receptors and for determining the host range of SARS-CoV-1 like viruses. Based on structural studies and biochemical experiments, SARS-CoV-2 seems to have an RBD that binds with high affinity to ACE2 also from ferrets and cats [9].

The WHO defines a confirmed case as “a person with laboratory confirmation of COVID-19 infection irrespective of clinical signs and symptoms”. Indeed, another issue to face, in the eye of the storm, was the capacity to perform thousands of tests per day. It is reasonable to understand that reliable and fast diagnosis of COVID-19 infection is a critical task to be performed. Without accurate collection of data and metadata on COVID-19 spread we cannot possibly understand how the pandemic is progressing. In this perspective, to support the SSN and to minimize the impact of this rapidly spreading virus, the Italian Ministry of Health (MoH) involved the Istituti Zooprofilattici Sperimentali (IZSs) in the diagnosis of SARS-CoV-2 by testing human samples. IZSs are Public Health institutes which are coordinated by the MoH and act as technical and operative support of the National Health Care System with regard to animal health, healthiness and quality control for foods of animal origin, breeding hygiene and correct relation between human and animal settlements and the environment. They are ten and represent a network throughout the entire National territory.

## 2 Materials and methods

This paper aims at describing the first three weeks of experience gained by the Istituto Zooprofilattico Sperimentale dell' Abruzzo e del Molise (IZSAM) in the melieu of COVID-19 crisis in support of the diagnostic workflow for SARS-CoV-2 of the Abruzzo region. The first case of COVID-19 in Abruzzo region was recorded on February 27^th^ in a male patient originating from Lombardy region who arrived in Abruzzo for tourism several days before the movement restrictions implemented first in Lombardy region and in other provinces of northern Italy, and then extended all over the Italian territory.

Samples tested for the presence of SARS-CoV-2 RNA are collected from the respiratory tract of individuals which are either hospitalized, or screened as for contact history with infected individuals or in the framework of the screening programs for workers of the SSN. For the vast majority, samples of hospitalized individuals originate from hospitals located in different cities of Abruzzo region: Teramo (Ospedale Civile Giuseppe Mazzini), Atri (Ospedale Civile S. Liberatore), Pescara (Ospedale Civile Spirito Santo, Pescara), Avezzano (Ospedale Civile SS. Nicola e Filippo), Sulmona (Ospedale SS Annunziata), Lanciano (Ospedale Renzetti), L'Aquila (Ospedale Regionale S. Salvatore) and Castel di Sangro (Ospedale Civile).

The workflow for SARS-CoV-2 RNA detection is composed by two steps. The first includes virus inactivation (PrimeStore® MTM, in BSL3 biocontainment laboratory) starting from a total volume of 200 μl of oropharyngeal (OF) swab transport medium (physiological solution) or bronchoalveolar lavage (BAL) and nucleic acid purification (MagMaxTM CORE) according to the manufacturer's instructions. The second consists of RNA detection by the TaqMan^TM^ 2019-nCoV Assay Kit v1 (Thermofisher, qPCR) whose results are interpreted following the manufacturer's instructions. Briefly, this test targets three different portions of SARS-CoV-2 genome located in the replicase, S and N protein encoding genes, respectively.

Laboratory activities are not limited to the molecular detection of SARS-CoV-2 RNA. Selected positive samples showing low threshold cycle (C_*T*_) values are regularly further processed by next generation sequencing (NGS) in order to obtain the whole genome sequence of the occurring strains. At the time this paper has been written, a total number of 46 samples were processed by NGS. They were selected within those collected from patients between the 16th and 23rd of March 2020.

RNA from these infected samples was treated with TURBO DNase (Thermo Fisher Scientific, Waltham, MA) at 37 °C for 20 min and then purified by RNA Clean and Concentrator-5 Kit (Zymo Research). RNA was used for the assessment of sequencing independent single primer amplification protocol (SISPA) with some modification [10]. Briefly, cDNA was obtained by reverse-transcription (RT) using SuperScript® IV Reverse Transcriptase (Thermo Fisher Scientific, Waltham, MA) and a combination of two primers including the random-tagged primer FR26RV-N 5’-GCCGGAGCTCTGCAGATATCNNNNNN-3′ with a poly-A tagged primer FR40RV-T 5’-GCCGGAGCTCTGCAGATATCTTTTTTTTTTTTTTTTTTTT-3′ [22]. The reaction was incubated at 23 °C for 10 min and at 50 °C for 50 min. After an inactivation step at 80 °C for 10 min, 2.5 units of Klenow Fragment (3′ → 5′ *exo*-) (New England Biolabs, Ipswich, MA) was directly added to the reaction to perform the second strand cDNA synthesis. The incubation was carried out at 37 °C for 1 h and 75 °C for 10 min. Next, 5 μl of the ds cDNA was added to PCR master mix containing 1× Q5 Reaction Buffer, Q5 High-Fidelity DNA Polymerase, dNTPs mix and the primer-tag FR20RV 5’-GCCGGAGCTCTGCAGATATC-3′ [11]. The incubation was performed with the following thermal conditions: 98 °C for 1 min, 40 cycles of 98 °C for 10 s, 65 °C for 30 s and 72 °C for 3 min and a final extension step of 72 °C for 2 min. The PCR product was purified by ExpinTM PCR SV (GeneAll Biotechnology CO., LTD Seoul, Korea) and then quantified using the QuantiFluor One ds DNA System kit (Promega). Libraries were prepared by using Nextera DNA Flex Library Prep (Illumina Inc., San Diego, CA) according to the manufacturer's protocol. Deep sequencing was performed on the MiniSeq (Illumina Inc., San Diego, CA) by the MiniSeq Mid Output Kit (300-cycles) and standard 150 bp paired-end reads. Reads obtained were trimmed by trimmomatic [12] and mapped on the host genome (GCF_000001405) using bowtie2 [13]; only unmapped reads were retained for downstream analysis. SARS-CoV-2 consensus sequence was obtained using samtools suite [14] after reads was mapped to reference sequence (NC_045512, Wuhan-Hu-1) by bowtie2.

## 3 Results

**Fig. 1:**
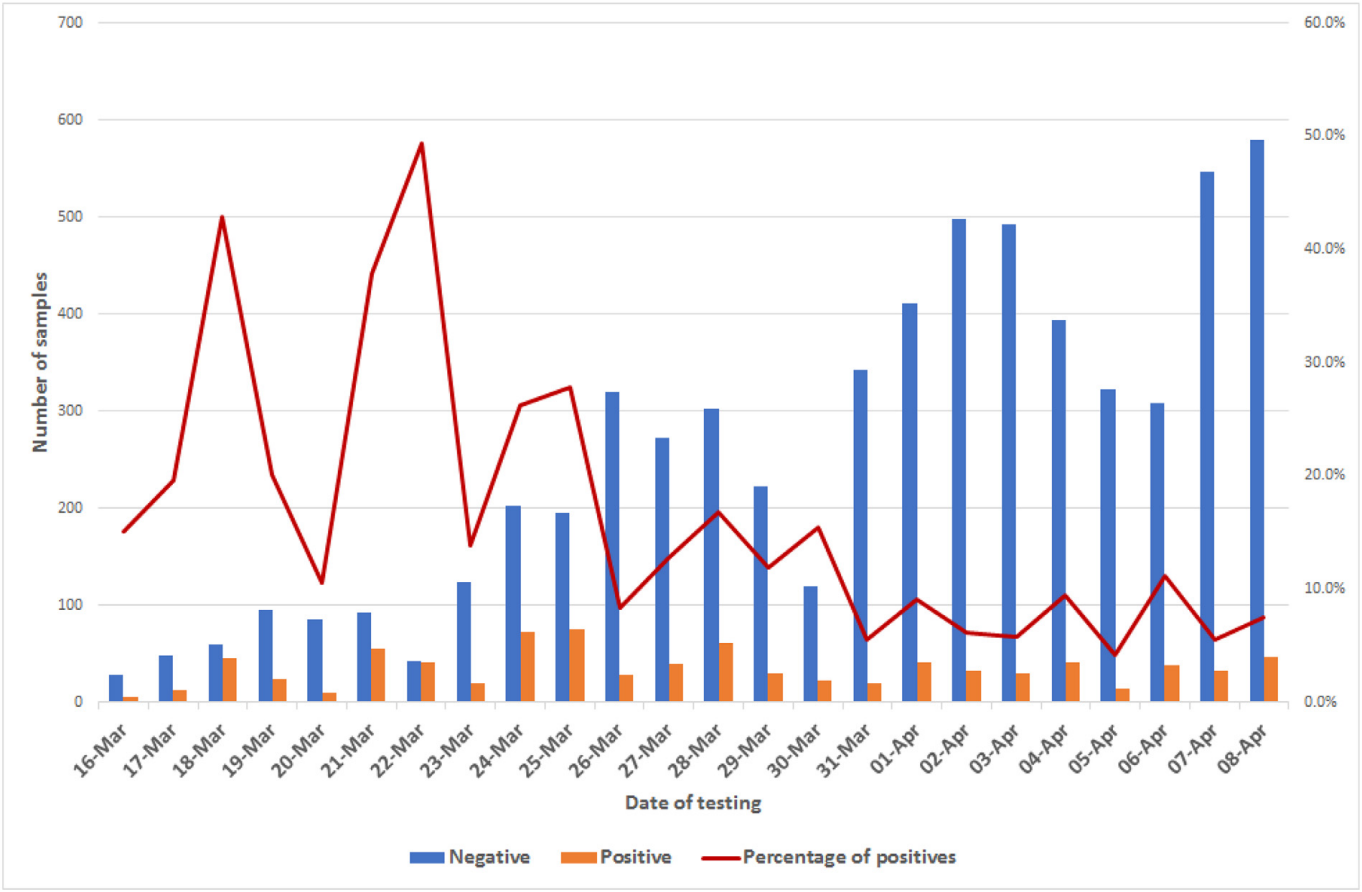
Temporal distribution of samples tested by results and percentage of positive samples.

**Fig. 2:**
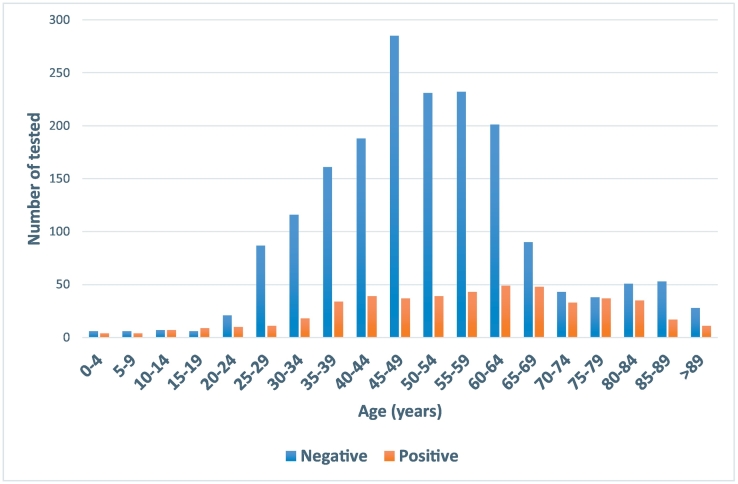
Number of positive and negative samples by age (years) of patients.

Starting from March 16^th^ and up to April 8^th^ around 8000 samples were processed at IZSAM. In the first week of testing, not more than 150–200 samples per day were tested, but in the following days the laboratory capacity was increased up to around 600 samples/day. Overall, 839 out of 7994 samples tested positive by qPCR (Fig. 1). Correlation between qPCR-negative/positive samples and age is showed in Table 1 and Fig. 2.

**Table 1:** Age mean values of individuals tested positive and negative for SARS-CoV-2. (*p*-Value <.0001, two tails Mann-Whitney Test).

	Age (years)	
	Negative	Positive
Mean	50.2	55.6
Median	49.6	56.9
Standard deviation	16.5	20.0

Out of 46 samples sent for NGS, 45/46 sequences were suitable for downstream analysis. Only one sequence was discarded as only few reads were obtained. Out of 45 sequences, 16 were complete or almost complete (horizontal coverage >95,2%) and with high vertical coverage. They were deposited in the GISAID database [15]; as listed in Table 2. All obtained sequences in this study showed >99% of nucleotide (nt) identity with Wuhan-Hu-1 (NC_045512) SARS-CoV-2 reference strain. However, all of them had single nucleotide polymorphisms (SNPs) with respect the reference Wuhan-Hu-1 sequence. All sequences either partial or complete, show a first common SNP mutation in the leader sequence (241C>T) which co-evolved with 3037C>T, 14408C>T, and 23403A>G [16]. While 3037C>T causes a synonymous mutations in nsp3 (F105F) 14408C>T and 23403A>G cause amino acid mutations in RNA primase (nsp 12, P323L), and S protein (D614G), respectively. The four co-mutations are prevalent in viral isolates from Europe. All sequences obtained in this study, but one, had 27046C (T175 in the coded M protein); one sequence from Pescara, which was not deposited with GISAID, had the mutation 27046C>T (T175M in the M protein). Moreover, 29/45 (12/16 of those which have been deposited) sequences showed R203K and G204R in the N protein as for the presence of mutations 28881G>A, 28882G>A, and 28883G>C in the nucleotide sequence. For 3/45 of sequences, the obtained sequence reads did not cover that portion of genome. According to GISAID (Genomic epidemiology of hCoV, https://www.gisaid.org/epiflu-applications/next-hcov-19-app/), these mutations in the N protein first appeared in a SARS-CoV-2 sequence from northern Europe (hCoV-19/Netherlands/Berlicum_1363564/2020, EPI_ISL_413565) originating from a sample collected on February 24^th^ with a travel history to Italy Regarding Italian sequences, the same mutations were also identified in one sequence recently released from the Laboratory of Virology Lazzaro Spallanzani (Rome) and collected on February 28^th^ from a male patient aged 41 years. Interestingly, a sequence obtained from a sample collected from the hospital of Atri (TE7097), which was not deposited with GISAID as for suboptimal horizontal coverage, did not show D614G in the S protein, typical of European strains, thus retaining the D614 of early Chinese strains. Unfortunately, we could not investigate for the presence of D614G co-mutations and residues in position 203 and 204 of the N protein as for the absence of sequence coverage in those portions of the genome. No mutations were observed in critical residues of the S1 protein.

**Table 2:** SARS-CoV-2 sequenced and deposited with GISAID. M, male; F, female. Age is expressed in years.

Strain	GISAID acc.no	Hospital	Sex, Age	N genotype	Vertical coverage	Horizontal coverage
hCoV-19/Italy/TE4836/2020	EPI_ISL_418260	Teramo	M, 41	R203, G204	346 X	98,90%
hCoV-19/Italy/TE4959/2020	EPI_ISL_418259	Pescara	M, 76	K203, R204	197 X	99,98%
hCoV-19/Italy/TE5056/2020	EPI_ISL_418257	Teramo	F, 75	K203, R204	297 X	99,99%
hCoV-19/Italy/TE4880/2020	EPI_ISL_418256	Atri	M, 80	K203, R204	1268 X	99,31%
hCoV-19/Italy/TE4925/2020	EPI_ISL_418255	Pescara	F, 63	K203, R204	1751 X	99,70%
hCoV-19/Italy/TE4953/2020	EPI_ISL_418258	Pescara	M, 87	K203, R204	2462 X	99,98%
hCoV-19/Italy/TE5052/2020	EPI_ISL_418261	Teramo	F, 78	R203, G204	87 X	95,19%
hCoV-19/Italy/TE5166/2020	EPI_ISL_420563	Teramo	M, 68	R203, G204	2501 X	100%
hCoV-19/Italy/TE5472/2020	EPI_ISL_420564	Castel di Sangro	M, 54	R203, G204	1960 X	99,98%
hCoV-19/Italy/TE5476/2020	EPI_ISL_420565	Teramo	M, 61	K203, R204	224 X	99,85%
hCoV-19/Italy/TE5512/2020	EPI_ISL_420566	L' Aquila	M, 71	K203, R204	101 X	99,67%
hCoV-19/Italy/6193/2020	EPI_ISL_420568	Teramo	M, 43	K203, R204	3721 X	99,87%
hCoV-19/Italy/TE6225/2020	EPI_ISL_420592	Teramo	F, 29	K203, R204	126 X	98,85%
hCoV-19/Italy/TE5780/2020	EPI_ISL_420567	L'Aquila	M, 64	K203, R204	447 X	99,86%
hCoV-19/Italy/TE6195/2020	EPI_ISL_420569	Teramo	M, 86	K203, R204	2850 X	99,97%
hCoV-19/Italy/TE6222/2020	EPI_ISL_420583	Teramo	M, 38	K203, R204	538 X	99,94%

Genome analysis suggests that different viral SARS-CoV-2 variants might be circulating in Italy and so in Abruzzo region.

Although the hallmark characterizing SARS-CoV-2 strains observed in this study are mainly located in the N protein, there is no evidence of geographical clustering in the Abruzzo region related to the two N protein viral variants. Sequences showing R203K and G204R in the N protein, according to GISAID, were evidenced primarily in northern Europe, but also recently in different countries including, within the others, USA, Spain, Greece, Vietnam and South America. As there is a critical lack of SARS-CoV-2 sequences from northern Italy, speculations upon the origin of the N protein viral variants can be made once a clearer picture of the genomic characteristics of the viruses circulating in Italy is available. In this regard, it would be important to obtain the sequence information of the early SARS-CoV-2 strains detected in Abruzzo and northern Italian regions to draw evidenced-based conclusions. The N protein of SARS-CoV-1 is responsible for the formation of the helical nucleocapsid during virion assembly. The N protein may cause an immune response and has potential value in vaccine development [17]. Hence, these mutations shall be considered when developing a vaccine using the N protein. Reasonably, the role of these mutations needs to be investigated by proper biochemical and reverse genetics experiments.

## 4 Discussion

Diagnosis of SARS-CoV-2 is currently performed in Italy and so in Abruzzo region, in a One Health perspective, with the support of the network of the IZSs. This decision arose by the combination of various relevant factors. Firstly, the IZSs belong to the SSN, coordinated by the MoH, and such condition facilitates the establishment of fruitful collaborations with the Public Health sectors, including the development of common diagnostic and data exchange protocols. Secondly, each IZS has the technical and scientific capacities to support the SSN to meet the extraordinary surge in demand for diagnostic testing of human samples for SARS-CoV-2. Lastly, IZSs have also experience in quality assurance, biosafety, biosecurity, and high throughput testing for the surveillance and control of infectious diseases in animals, some of which, including the current SARS-CoV-2, are zoonotic. Moreover, they are also equipped with large infrastructure for genomic analysis and storage of sequence data. These infrastructures are routinely used in animal health and food security emergences and for diagnostic purposes. In this regard, the analysis of the whole viral genome of SARS-CoV-2 strains is a critical task. However, still scarce is genomic data (and related metadata) available from SARS-CoV-2 strains circulating in Italy and further efforts are necessarily warranted. Whole genome sequencing straight from infected biological samples may indeed provide useful information to identify mutations during the virus adaptation to humans, such as mutations in critical residues of the S protein or resulting in the loss of accessory genes as already described for SARS-CoV-1 [18]. An additional factor which may have influenced the choice of appointing IZSs to support the SSN's effort against COVID-19 was related to the biological nature of the occurring agent. CoVs act as primary actors within the so-called human/animal interface across which a plethora of infectious pathogens has been observed to emerge, spill over various species and eventually evolve, thus finding new ecological niches and causing new epidemiological phenomena. Of value, in the Italian context, is certainly the experience that veterinarians operating within the Public Health system gained in the control and characterization of previous health issues of livestock and poultry including avian flu, bluetongue, foot and mouth disease, and BSE, which were responsible for huge economic losses. This aptitude of being “*ready to action*” during a health emergence certainly includes rapid diagnosis, epidemiological investigations, molecular/antigenic characterization, development of vaccines, and planning of surveillance programs, a process that is pursued, together with saving patients' lives in hospitals, by technicians and scientists around the globe for COVID-19. We add to this the fact that veterinarians have known of CoVs and related diseases for decades [5,19], thus, the One Health concept is central and should again be sublimated and adopted to control critical health emergencies, including that of antimicrobial resistance.

Therefore, the multidisciplinary involvement of different professionals operating within the SSN is crucial to properly and effectively face the challenges posed by viruses like SARS-CoV-2. A holistic and One Health approach is the sole solution for better understanding the epidemiological aspect of this disease and possibly preventing the establishment of new transmission chains. Currently, the available genome sequences so far clearly reveal that the most closely related virus (96.2% of nt sequence identity) to SARS-CoV-2 is a strain from a bat, *Rhinolophus affinis*, identified as strain BatCoVRaTG13 from a faecal sample in Yunnan province, China; and that the next closest viruses are SARS-rCoVs identified from pangolins [23], however, the exact origin of SARS-CoV-2 has yet to be demonstrated. In this perspective, veterinary virologists may surely support this important task as well as those doomed to understand SARS-CoV-2 virulence factors through the assessment of reverse genetics studies and animal models, and to analyze the impact of the hyperinflammation observed in COVID-19 infected patients, characterized by a cytokine storm. This latter evidence is not novel for veterinarians as it is observed in cats infected with feline infectious peritonitis virus, a lethal pathotype of the feline enteric coronavirus [20].

As for cats, recent evidences also demonstrated that they might get infected from COVID-19 positive humans (https://www.nature.com/articles/d41586-020-00984-8) or following experimental infection [21], thus confirming the high affinity of SARS-CoV-2 with feline ACE2. Although the role of domestic animals in the epidemiology of SARS-CoV-2 seems to be negligible, further studies are reasonably warranted. Moreover, to plan future strategies for SARS-CoV-2 containment, it will be essential to better understand the protective role of the various classes of antibodies against the virus, as well as the prevalence of serologically positive individuals in the human population when the epidemic curve has shown a stable decrease. Also for these purposes, the IZSs' laboratories will be useful for processing a large number of serum samples and to support the Public Health Institutes in the necessary experimental studies.

## Declaration of Competing Interest

Authors declare no conflict of interest.

## References

[1] Zhou P., Yang X.L., Wang X.G., Hu B., Zhang L., Zhang W., Si H.R., Zhu Y., Li B., Huang C.L., Chen H.D., Chen J., Luo Y., Guo H., Jiang R.D., Liu M.Q., Chen Y., Shen X.R., Wang X., Zheng X.S., Zhao K., Chen Q.J., Deng F., Liu L.L., Yan B., Zhan F.X., Wang Y.Y., Xiao G.F., Shi Z.L. (2020). A pneumonia outbreak associated with a new coronavirus of probable bat origin. Nature.

[2] Gorbalenya A.E., Baker S.C., Baric R.S., de Groot R.J., Drosten C., Gulyaeva A.A., Haagmans B.L., Lauber C., Leontovich A.M., Neuman B.W., Penzar D., Perlman S., Poon L.L.M., Samborskiy D.V., Sidorov I.A., Sola I., Ziebuhr J. (2020). The species Severe acute respiratory syndrome-related coronavirus: classifying 2019-nCoV and naming it SARS-CoV-2. Nat. Microbiol..

[3] Yang X., Yu Y., Xu J., Shu H., Xia J., Liu H. (2020). Clinical course and outcomes of critically ill patients with SARS-CoV-2 pneumonia in Wuhan, China: a single-centered, retrospective, observational study. Lancet Respir. Med..

[4] Matthay M.A., Zemans R.L., Zimmerman G.A. (2019). Acute respiratory distress syndrome. Nat. Rev. Dis. Primers.

[5] Lorusso A., Calistri P., Petrini A., Savini G., Decaro N. (2020). Novel coronavirus (SARS-CoV-2) epidemic: a veterinary perspective. Vet. Ital..

[6] Li T., Zhang Y., Fu L., Yu C., Li X., Li Y., Zhang X., Rong Z., Wang Y., Ning H. (2005). siRNA targeting the leader sequence of SARS-CoV inhibits virus replication. Gene Ther..

[7] Ziebuhr J., Snijder E.J., Gorbalenya A.E. (2000). Virus-encoded proteinases and proteolytic processing in the Nidovirales. J. Gen. Virol..

[8] Walls A.C., Park Y.J., Tortorici M.A., Wall A., McGuire A.T., Veesler D. (2020). Structure, function, and antigenicity of the SARS-CoV-2 spike glycoprotein. Cell.

[9] Andersen K.G., Rambaut A., Lipkin W.I. (2020). The proximal origin of SARS-CoV-2. Nat. Med..

[10] Marcacci M., De Luca E., Zaccaria G., Di Tommaso M., Mangone I., Aste G., Savini G., Boari A., Lorusso A. (2016). Genome characterization of feline morbillivirus from Italy. J. Virol. Methods.

[11] Allander T., Tammi M.T., Eriksson M., Bjerkner A., Tiveljung-Lindell A., Andersson B. (2005). Cloning of a human parvovirus by molecular screening of respiratory tract samples. Proc. Natl. Acad. Sci. U. S. A..

[12] Bolger A.M., Lohse M., Usadel B. (2014). Trimmomatic: a flexible trimmer for illumina sequence data. Bioinformatics.

[13] Langmead B., Salzberg S. (2012). Fast gapped-read alignment with bowtie 2. Nat. Methods.

[14] Li H., Handsaker B., Wysoker A., Fennell T., Ruan J., Homer N., Marth G., Abecasis G., Durbin R., 1000 Genome Project Data Processing Subgroup (2009). The Sequence alignment/map (SAM) format and SAMtools. Bioinformatics.

[15] Shu Y., McCauley J. (2017). GISAID: Global initiative on sharing all influenza data – from vision to reality. EuroSurveillance.

[16] Yin C. (2020). Genotyping Coronavirus SARS-CoV-2: Methods and implications..

[17] Zhao P., Cao J., Zhao L., Qin Z., Ke J., Pan W., Ren H., Yu J., Qi Z. (2005). Immune responses against SARS-coronavirus nucleocapsid protein induced by DNA vaccine. Virology.

[18] Cui J., Li F., Shi Z.L. (2019). Origin and evolution of pathogenic coronaviruses. Nat. Rev. Microbiol..

[19] Decaro N., Lorusso A. (2020). Novel human coronavirus (SARS-CoV-2): a lesson from animal coronaviruses. Vet. Microbiol..

[20] Decaro N., Martella V., Saif L.J., Buonavoglia C. (2020). COVID-19 from veterinary medicine and one health perspectives: what animal coronaviruses have taught us. Res. Vet. Sci..

[21] Shi J., Wen Z., Zhong G., Yang H., Wang C., Liu R., He X., Shuai L., Sun Z., Zhao Y., Liang L., Cui P., Wang J., Zhang X., Guan Y., Chen H., Bu Z. (2020). Susceptibility of ferrets, cats, dogs, and other domesticated animals to SARS-coronavirus 2. Science.

[22] Djikeng A., Halpin R., Kuzmickas R., Depasse J., Feldblyum J., Sengamalay N., Afonso C., Zhang X., Anderson N.G., Ghedin E., Spiro D.J. (2008). Viral genome sequencing by random priming methods. BMC Genomics.

[23] Lam T.T., Shum M.H., Zhu H.C., Tong Y.G., Ni X.B., Liao Y.S., Wei W., Cheung Y.W., Li W.J., Li L.F., Leung G.M., Holmes E.C., Hu Y.L., Guan Y. (2020). Identifying SARS-CoV-2 related coronaviruses in Malayan pangolins. Nature.

